# Supplementary material for: Psychometric Validation of the German Translation of the Quality of Life Questionnaire-Bronchiectasis (QOL-B)—Data from the German Bronchiectasis Registry PROGNOSIS
Source: J Clin Med. 2022 Jan 15;11(2):441. doi: 10.3390/jcm11020441 (PMC8781204; doi:10.3390/jcm11020441)
Supplement: Supplementary file 1 [file jcm-11-00441-s001.zip › Quellhorst_JCM_Supplementary_file_S1_05-Dec-2021.pdf]

Psychometric Validation of the German Translation of the Quality of Life Questionnaire Bronchiectasis (QOL-B) – Data from the German Bronchiectasis Registry PROGNOSIS

Laura Quellhorst, Grit Barten, Andrés de Roux, Roland Diel, Pontus Mertsch, Isabell Pink, Jessica Rademacher, Sivagurunathan Sutharsan, Tobias Welte, Annegret Zurawski, and Felix C. Ringshausen, and the PROGNOSIS study group

SUPPLEMENTAL FILE 1 (S1)

SAS and SPSS Programming Code for Scoring QOL-B V3.1

Note: To enter data, refer to **Step 1: Item-by-item responses,** and for decision rules regarding multiple responses or skipped questions, refer to **Step 2: Scoring multiple responses or skipped questions** (see page 1).

SAS Program Codes for Scoring the QOL-B

```
/*This scoring program requires that the data be imported into a SAS table titled "QOLB"
and that the variable names in the table match those listed below.*/

Data QOLB; set QOLB;

/* Rescaling Respiratory 32 */
if resp32=6 then resp32=.;
else if resp32=1 then resp32= 4;
else if resp32=2 then resp32= 3;
else if resp32=3 then resp32= 2;
else if resp32=4 then resp32= 1;
else if resp32=5 then resp32= 1;

/* Rescaling “doesn’t apply” Social 19 */
if social19=5 then social19=.;

/* Recoding Some Variables */
health5=5-health5;
vital8=5-vital8;
treat12=5-treat12;
treat14=5-treat14;
health15=5-health15;
role20=5-role20;
health24=5-health24;
role27=5-role27;
run;


/* Calculating Scores */

Data QOLB; set QOLB;
```

```
if nmiss (phys1, phys2, phys3, phys4, phys16) <= 2 then
physical = (mean (phys1, phys2, phys3, phys4, phys16)-1)/3*100;

if nmiss (role17, role20, role25, role27, role28) <= 2 then
role = (mean (role17, role20, role25, role27, role28)-1)/3*100;

if nmiss (vital6, vital8, vital9) <= 1 then
vitality = (mean (vital6, vital8, vital9)-1)/3*100;

if nmiss (emot7, emot10, emot11, emot23) <= 2 then
emotion = (mean (emot7, emot10, emot11, emot23)-1)/3*100;

if nmiss (social18, social19, social22, social26) <= 2 then
social = (mean (social18, social19, social22, social26)-1)/3*100;

if nmiss (treat12, treat13, treat14) <= 1 then
treat = (mean (treat12, treat13, treat14)-1)/3*100;

if nmiss (health5, health15, health21, health24) <= 2 then
health = (mean (health5, health15, health21, health24)-1)/3*100;

if nmiss (resp29, resp30, resp31, resp32, resp33, resp34, resp35, resp36, resp37) <= 4
then
respirat = (mean (resp29, resp30, resp31, resp32, resp33, resp34, resp35, resp36,
resp37)-1)/3*100;
run;
```

SPSS Program Codes for Scoring the QOL-B Version 3.1

Note: To enter data, refer to **Step 1: Item-by-item responses,** and for decision rules regarding multiple responses or skipped questions, refer to **Step 2: Scoring multiple responses or skipped questions** (see page 1).

```
*Recoding Some Items.

recode health5 vital8 treat12 treat14 health15 role20 health24 role27 (1=4) (2=3) (3=2)
(4=1).
Execute.

recode social19 (5=SYSMIS).
Execute.

recode resp32 (1=4) (2=3) (3=2) (4=1) (5=1) (6=SYSMIS).
Execute.

 *Calculating Scores.
compute physical = (mean.2 (phys1, phys2, phys3, phys4, phys16)-1)/3*100.
compute role = (mean.2 (role17, role20, role25, role27, role28)-1)/3*100.
compute vitality = (mean.1 (vital6, vital8, vital9)-1)/3*100.
compute emotion = (mean.2 (emot7, emot10, emot11, emot23)-1)/3*100.
compute social = (mean.2 (social18, social19, social22, social26)-1)/3*100.
compute treat = (mean.1 (treat12, treat13, treat14)-1)/3*100.
compute health = (mean.2 (health5, health15, health21, health24)-1)/3*100.
compute respirat = (mean.4 (resp29, resp30, resp31, resp32, resp33, resp34, resp35,
resp36, resp37)-1)/3*100.
Execute.
```

Manual Scoring Instructions for QOL-B Version 3.1

Step 1: Item-by-item responses

The values assigned to participants' responses for each question are listed below. Enter them on the Item-by-Item Worksheet.

- For questions 1 – 4: A lot of difficulty = 1, Moderate difficulty = 2, A little difficulty = 3, No difficulty = 4
- For questions 5 – 11: Always = 1, Often = 2, Sometimes = 3, Never = 4
- For questions 12 – 15: Use the assigned number designated for each specific response
- For questions 16 – 26: Completely true = 1, Mostly true = 2, A little true = 3, Not at all true = 4
- For question 27: Use the assigned number designated for each specific response
- For question 28: Always = 1, Often = 2, Sometimes = 3, Never = 4
- For questions 29 – 31: A lot = 1, A moderate amount = 2, A little = 3, Not at all = 4
- For question 32: Clear = 1, Clear to yellow = 2, Yellowish-green = 3, Brownish-dark = 4, Green with traces of blood = 4, Don't know = 6
- For questions 33 – 37: Always = 1, Often = 2, Sometimes = 3, Never = 4

Step 2: Scoring multiple responses or skipped questions

If two responses are marked and there is no opportunity to ask the respondent which one is correct, the **worst response** should be selected for data entry and scoring. This provides a conservative estimate of their response to this item. For example, item #29 asks: "Have you felt congestion in your chest?" The response choices range from "A lot" to "Not at all." If the respondent marks "a lot" and "a moderate amount" you should enter "a lot" for this question.

Please note that some items are reverse-keyed and therefore, the worst response is not necessarily the lower number.

If participants skip a question, do not assign a response value (i.e. leave it blank).

Step 3: Scaling item 32 and reverse coding

Item 32 (resp32) has 5 possible answers that are scored and all other items on the QOL-B questionnaire have only 4 possible answers. Possible scores for resp32 are 1, 2, 3, 4, 5 and 6, whereas for other questions the possible scores are 1, 2, 3, and 4. Resp32 and eight other items are also reverse coded; because of the wording for these particular items, reverse coding is necessary to make higher scores correspond to better health outcomes. Reverse coding is conducted for resp32, and for health5, vital8, treat12, treat14, health15, role20, health24, and role27. These items are marked with an asterisk on the Item-by-Item Worksheet and the reverse-coded values are shown in the box on the worksheet.

For item 32:

Original value = Reverse-coded value
1 = 4
2 = 3
3 = 2
4 = 1
6 = Not scored

For item 19:

"doesn't apply" = Not scored

For items 5, 8, 12, 14, 15, 20, 24, 27:

Original value = Reverse-coded value
1 = 4
2 = 3
3 = 2
4 = 1

Step 4: Preparing to calculate scaled scores and missing values

Transfer the values from the Item-by-Item Worksheet to the Scaled Score Worksheet. For reverse-coded items, use the reverse-coded values. Do not enter any values for missing responses; leave the line blank. If the responses are missing for more than half the items in a scale, the score for that scale should not be calculated. Missing values are not imputed. Note that missing responses within a scale will change the number of points corresponding to a change of one answer category for one item for that respondent.

Step 5: Calculate the scaled scores

Calculate scores for the eight QOL-B domains using the formulas on the Scaled Score Worksheet. Note that a total QOL-B score is not calculated.

Item-by-Item Worksheet

Numbers correspond to items on the QOL-B Version 3.1 questionnaire. Fill in the values using the scoring rules described in steps 1 – 3.

1. _____
2. _____
3. _____
4. _____
5.* _____=_____
6. _____
7. _____
8.* _____=_____
9. _____
10. _____
11. _____
12.*_____=_____
13. _____
14.*_____=_____
15.*_____=_____
16. _____
17. _____
18. _____
19. _____
20.*_____=_____
21. _____
22. _____
23. _____
24.*_____=_____
25. _____
26. _____
27.*_____=_____
28. _____
29. _____
30. _____
31. _____
32.*_____=_____
33. _____
34. _____
35. _____
36. _____
37. _____

***Reverse-Coded Values**

For items 5, 8, 12, 14, 15, 20, 24, 27:

Original Value = Reverse-Coded Value
1 = 4
2 = 3
3 = 2
4 = 1

For item 32:

Original Value = Reverse-Coded Value
1 = 4
2 = 3
3 = 2
4 = 1
6 = Not scored

Enter the values from the Item-by-Item Worksheet. Use the reverse-coded values, if applicable. Do not enter any values for missing responses; leave the line blank. Assess the number of missing values and calculate scores as described in Steps 4 and 5 (see page 2).

Physical Functioning Domain (5 items)

1. _____
2. _____
3. _____
4. _____
16. _____

If 3 or more responses are missing, do not score this domain.
Scaled score = [((_______________)-1)/3] x 100 = _______
mean of responses

Role Functioning Domain (5 items)

17. _____
20. _____
25. _____
27. _____
28. _____

If 3 or more responses are missing, do not score this domain.
Scaled score = [((_______________)-1)/3] x 100 = _______
mean of responses

Vitality Domain (3 items)

6. _____
8. _____
9. _____

If 2 or more responses are missing, do not score this domain.
Scaled score = [((_______________)-1)/3] x 100 = _______
mean of responses

Emotional Functioning Domain (4 items)
7. _____
10. _____
11. _____
23. _____

If 3 or more responses are missing, do not score this domain.
Scaled score = [((_______________)-1)/3] x 100 = _______
mean of responses

Social Functioning Domain (4 items)
18. _____
19. _____ (* "doesn't apply" = Not scored)
22. _____
26. _____

If 3 or more responses are missing, do not score this domain.
Scaled score = [((_______________)-1)/3] x 100 = _______
mean of responses

Treatment Burden Domain (3 items)
12. _____
13. _____
14. _____

If 2 or more responses are missing, do not score this domain.
Scaled score = [((_______________)-1)/3] x 100 = _______
mean of responses

Health Perceptions Domain (4 items)
5. _____
15. _____
21. _____
24. _____

If 3 or more responses are missing, do not score this domain.
Scaled score = [((_______________)-1)/3] x 100 = _______
mean of responses

Respiratory Symptoms Domain (9 items)

29. _____
30. _____
31. _____
32. _____
33. _____
34. _____
35. _____
36. _____
37. _____

If 5 or more responses are missing, do not score this domain.

Scaled score = [((________________)-1)/3] x 100 = _______
mean of responses

Note: No total score is calculated.

Psychometric Validation of the German Translation of the Quality of Life Questionnaire Bronchiectasis (QOL-B) – Data from the German Bronchiectasis Registry PROGNOSIS

Laura Quellhorst, Grit Barten, Andrés de Roux, Roland Diel, Pontus Mertsch, Isabell Pink, Jessica Rademacher, Sivagurunathan Sutharsan, Tobias Welte, Annegret Zurawski, and Felix C. Ringshausen, and the PROGNOSIS study group

SUPPLEMENTARY MATERIALS 2 (S2)

Supplementary Table S2.1. Comparison of demographic and baseline characteristics between all subjects enrolled in PROGNOSIS until Mach 2018 (N=1000) and those who completed the QOL-B (N=904)

Variable		Value	Value
**N (total)**		**904**	**1000**
Age (years), mean (SD)		59.5 (15.8)	59.3 (15.8)
	<50 years, n (%)	215 (23.8)	247 (24.7)
	50-69 years, n (%)	401 (44.4)	442 (44.2)
	70-79 years, n (%)	253 (28.0)	273 (27.3)
	≥80 years, n (%)	35 (3.9)	38 (3.8)
Females, n (%)		538 (59.5)	593 (59.3)
BMI (kg/m^2), mean (SD)		24.1 (4.5)	24.1 (4.6)
	<18.5 kg/m^2	80 (8.8)	88 (8.8)
	18.5-30 kg/m^2	740 (81.9)	822 (82.2)
	>30 kg/m^2	84 (9.3)	90 (9.0)
FEV_1 %predicted, mean (SD)		69.8 (26.8)	69.4 (26.6)
	<30 %predicted, n (%)	58 (6.4)	63 (6.3)
	30-49 %predicted, n (%)	180 (19.9)	202 (20.2)

	50-79 %predicted, n (%)	322 (35.6)	360 (36.0)
	≥80 %predicted, n (%)	344 (38.1)	375 (37.5)
Etiology, n (%)			
	Idiopathic	337 (37.3)	358 (35.8)
	Postinfectious/posttuberculous	180 (19.9)	212 (21.2)
	COPD	133 (14.7)	149 (14.9)
	Asthma	99 (11.0)	111 (11.1)
	Primary Ciliary Dyskinesia / Kartagener syndrome	79 (8.7)	88 (8.8)
	Other	76 (8.4)	82 (8.2)
Radiological Severity			
	<3 lobes affected	325 (36.0)	371 (37.1)
	≥3 lobes affected	422 (46.7)	463 (46.3)
	Cystic bronchiectasis	157 (17.4)	166 (16.6)
MRC dyspnea scale, n(%)			
	1	228 (25.2)	262 (26.2)
	2	301 (33.3)	339 (33.9)
	3	222 (24.6)	236 (23.6)
	4	108 (11.9)	116 (11.6)
	5	45 (5.0)	47 (4.7)
	1-3	751 (83.1)	837 (83.7)
	4-5	153 (16.9)	163 (16.3)
Smoking, n (%)			
	Active smoker	57 (6.3)	65 (6.5)
	Former smoker	335 (37.1)	371 (37.2)
	Never smoked	512 (56.6)	560 (56.2)
Exacerbations in the past 12 months, median (IQR)		1 (0-3)	1 (0-3)
	0, n (%)	271 (30.0)	304 (30.4)
	1-2, n (%)	361 (39.9)	394 (39.4)
	≥3, n (%)	272 (30.1)	302 (30.2)
Hospitalizations in the past 12 months, median (IQR)		0 (0-1)	0 (0-1)
Prior hospital admission, n (%)		349 (38.6)	387 (38.7)
Regular pharmacological treatment of bronchiectasis		704 (77.9)	766 (76.6)
Prior thoracic surgery		79 (8.7)	90 (9.0)
Regular sputum production, n (%)		704 (77.9)	780 (78.0)
Average daily sputum volume, median (IQR)		20 (10-50)	20 (10-50)

	0 mL/day, n (%)	284 (31.4)	311 (31.1)
	1-10 mL/day, n (%)	261 (28.9)	284 (28.4)
	11-20 mL/day, n (%)	122 (13.5)	128 (12.8)
	21-50 mL/day, n (%)	151 (16.7)	180 (18.0)
	51-100 mL/day, n (%)	60 (6.6)	67 (6.7)
	>100 mL/day, n (%)	26 (2.9)	30 (3.0)
QoL-B scales, mean (SD)			
	Respiratory Symptoms (n=892)	56.2 (21.0)	
	Physical Functioning (n=889)	41.8 (29.8)	
	Vitality (n=892)	42.0 (21.4)	
	Role Functioning (n=898)	58.8 (27.4)	
	Health Perceptions (n=891)	36.3 (22.6)	
	Emotional Functioning (n=889)	69.2 (21.9)	
	Social Functioning (n=878)	59.9 (26.9)	
	Treatment Burden (n=645) [1]	51.3 (25.1)	
Microbiology		n = 680	n = 756
	Pseudomonas aeruginosa, n (%)	223 (32.8)	249 (32.9)
	Staphylococcus aureus, n (%)	112 (16.5)	124 (16.4)
	Haemophilus influenzae, n (%)	93 (13.7)	103 (13.6)
	Aspergillus fumigatus, n (%)	73 (10.7)	79 (10.4)
	Nontuberculous mycobacteria, n (%)	41 (6.0)	48 (6.3)
BSI		n = 666	n = 736
	Mild (0–4)	150 (22.5)	171 (23.2)
	Moderate (5–8)	390 (58.6)	436 (59.2)
	Severe (≥9)	126 (18.9)	129 (17.5)
Comorbidities			
	Cardiovascular	354 (39.2)	383 (38.3)
	COPD	269 (29.8)	307 (30.7)
	Asthma	269 (29.8)	293 (29.3)
	Chronic rhinosinusitis	270 (29.9)	293 (29.3)
	Gastro-esophageal reflux (self-reported)	166 (18.4)	188 (18.8)
	Nasal polyps	141 (15.6)	155 (15.5)
	Malignancy	108 (11.9)	117 (11.7)
	Osteoporosis	98 (10.8)	104 (10.4)
	Depression	86 (9.5)	99 (9.9)

	Diabetes	79 (8.7)	88 (8.8)
	Renal insufficiency	71 (7.9)	73 (7.3)
	Anxiety disorder	34 (3.8)	39 (3.9)
	Liver cirrhosis	9 (1.0)	11 (1.1)

[1] Patients not receiving bronchiectasis treatment were instructed to skip the Treatment Burden scale. Abbreviations: BMI, body mass index; BSI, Bronchiectasis Severity Index; COPD, chronic obstructive pulmonary disease; FEV_1, forced expiratory volume in 1 second; IQR, interquartile range; MRC, Medical Research Council; QOL-B, QOL-B, Quality of Life Questionnaire-Bronchiectasis; PCD, primary ciliary dyskinesia; SD, standard deviation.

Supplementary Table S2.2. Discrimination of QOL-B scores, stratified by MRC dyspnea scale

**Mean (SD) QOL-B scores at baseline according to MRC dyspnea scale**						
**QOL-B scale**	**1**	**2**	**3**	**4**	**5**	***p*-Value** [1]
Respiratory Symptoms	69.5 (19.0)	56.9 (17.7)	49.6 (18.4)	42.1 (17.9)	42.4 (21.2)	<0.001
Physical Functioning	67.4 (27.4)	44.5 (23.5)	27.8 (18.9)	15.9 (18.5)	12.3 (21.8)	<0.001
Vitality	51.5 (21.3)	43.1 (20.7)	38.9 (18.9)	31.0 (18.5)	29.0 (22.4)	<0.001
Role Functioning	73.9 (23.3)	64.1 (23.4)	52.9 (22.2)	32.9 (21.7)	25.5 (21.4)	<0.001
Health Perceptions	51.0 (23.9)	37.9 (19.9)	29.0 (18.0)	22.7 (16.4)	19.0 (16.4)	<0.001
Emotional Functioning	74.9 (20.7)	71.8 (20.7)	66.1 (20.5)	59.3 (20.8)	53.3 (26.7)	<0.001
Social Functioning	66.3 (26.8)	62.1 (22.8)	54.4 (27.4)	48.9 (26.3)	47.8 (26.3)	<0.001
Treatment Burden	66.3 (22.7)	54.8 (22.9)	44.6 (23.0)	36.3 (23.1)	32.9 (21.8)	<0.001

[1] Differences between groups were assessed by the Kruskal-Wallis test. Abbreviations: MRC, Medical Research Council; QOL-B, Quality of Life Questionnaire-Bronchiectasis; SD, standard deviation.

Supplementary Table S2.3. Discrimination of QOL-B scores, stratified by BSI categories (n = 666)

**Mean (SD) QOL-B scores at baseline according to BSI category**				
**QOL-B scale**	**Mild (0–4)**	**Moderate (5–8)**	**Severe (≥9)**	***p*-Value** [1]
Respiratory Symptoms	61.8 (18.7)	52.3 (21.5)	51.4 (18.0)	<0.001
Physical Functioning	56.9 (27.6)	35.6 (28.7)	32.5 (26.0)	<0.001
Vitality	46.2 (20.9)	40.0 (21.9)	38.9 (19.5)	0.002
Role Functioning	72.5 (21.4)	53.7 (27.3)	46.5 (26.1)	<0.001
Health Perceptions	43.2 (22.4)	31.8 (21.3)	29.9 (19.4)	<0.001
Emotional Functioning	72.8 (20.2)	66.6 (22.8)	69.7 (20.4)	0.025
Social Functioning	61.8 (25.1)	57.0 (27.8)	53.5 (26.3)	0.033
Treatment Burden	55.1 (23.6)	46.9 (24.6)	45.1 (22.1)	0.006

[1] Differences between groups were assessed by the Kruskal-Wallis test. Abbreviations: BSI, Bronchiectasis Severity index; QOL-B, Quality of Life Questionnaire-Bronchiectasis; SD, standard deviation.

Supplementary Table S2.4. Discrimination of QOL-B scores, stratified by $ppFEV_1$ (categorized)

Mean (SD) QOL-B scores at baseline according to $ppFEV_1$					
**QOL-B scale**	**≥80**	**50-79**	**30-49**	**<30**	***p*-Value** [1]
Respiratory Symptoms	63 (18.8)	54.0 (20.2)	50.2 (19.1)	42.6 (18.6)	<0.001
Physical Functioning	55.5 (28.7)	40.4 (26.1)	25.3 (22.3)	15.2 (16.4)	<0.001
Vitality	45.2 (21.5)	41.5 (21.3)	41.5 (19.9)	31.9 (19.5)	<0.001
Role Functioning	66.3 (24.1)	59.5 (24.0)	51.1 (27.7)	33.4 (27.0)	<0.001
Health Perceptions	42.2 (23.6)	36.2 (20.7)	30.3 (20.4)	25.4 (19.2)	<0.001
Emotional Functioning	71.1 (21.3)	70.3 (20.4)	67.4 (22.3)	58.5 (23.2)	0.003
Social Functioning	60.5 (25.7)	57.4 (27.4)	60.1 (25.8)	51.7 (25)	0.087
Treatment Burden	59.2 (24.3)	49.9 (24.8)	46.6 (23.5)	38.8 (23.9)	<0.001

[1] Differences between groups were assessed by the Kruskal-Wallis test. Abbreviations: $ppFEV_1$, forced expiratory volume in one second (% predicted); QOL-B, Quality of Life Questionnaire-Bronchiectasis; SD, standard deviation.

Supplementary Table S2.5. Discrimination of QOL-B scores, stratified by average daily sputum volume (categorized)

Mean (SD) QOL-B scores at baseline according to average daily sputum volume (mL/day)							
**QOL-B scale**	**0**	**1-10**	**11-20**	**21-50**	**51-100**	**>100**	***p*-Value** [1]
Respiratory Symptoms	61.9 (22.6)	57.0 (19.4)	52.1 (17.8)	50.8 (18.1)	43.1 (17.4)	39.8 (14.7)	<0.001
Physical Functioning	44.5 (31.4)	42.0 (28.4)	37.8 (26.3)	37.7 (28.4)	30.7 (24.4)	27.0 (23.5)	0.001
Vitality	44.0 (22.1)	43.4 (21.0)	39.5 (19.7)	41.4 (20.2)	34.0 (20.1)	38.2 (23.7)	0.059
Role Functioning	62.3 (27.8)	57.4 (28.0)	55.8 (21.9)	57.3 (26.8)	44.2 (27.0)	52.1 (20.5)	<0.001
Health Perceptions	41.4 (23.4)	36.8 (22.9)	32.5 (19.2)	33.5 (20.8)	24.1 (19.0)	23.4 (15.0)	<0.001
Emotional Functioning	70.5 (22.3)	69.3 (20.9)	67.3 (20.0)	68.8 (21.9)	62.1 (21.3)	62.0 (21.9)	0.056
Social Functioning	65.1 (26.5)	60.1 (25.2)	55.5 (25.0)	55.1 (26.3)	43.3 (27.6)	46.0 (23.4)	<0.001
Treatment Burden	58.8 (24.4)	52.1 (24.9)	49.4 (23.7)	46.8 (24.0)	39.0 (24.3)	35.4 (22.7)	<0.001

[1] Differences between groups were assessed by the Kruskal-Wallis test. Abbreviation: QOL-B, Quality of Life Questionnaire-Bronchiectasis; SD, standard deviation.

Supplementary Table S2.6. Discrimination of QOL-B scores, stratified by regular pharmacological treatment of bronchiectasis

Mean (SD) QOL-B scores for patients with regular pharmacological treatment			
**QOL-B scale**	**Yes**	**No**	***p*-Value** [1]
Respiratory Symptoms	54.3 (20.6)	62.7 (21.4)	<0.001
Physical Functioning	40.1 (29.0)	48.1 (31.9)	0.003
Vitality	41.7 (21.4)	43.2 (21.4)	0.422
Role Functioning	57.2 (27.4)	64.7 (26.6)	0.001
Health Perceptions	35.2 (22.3)	40.2 (23.2)	0.008
Emotional Functioning	69.1 (21.9)	69.5 (21.7)	0.906
Social Functioning	57.8 (27.1)	67.0 (24.9)	<0.001
Treatment Burden	49.5 (25.0)	61.2 (23.4)	<0.001

[1] Differences between groups were assessed by the Mann-Whitney U test. Abbreviations: QOL-B, Quality of Life Questionnaire-Bronchiectasis; SD, standard deviation.

Supplementary Table S2.7. Discrimination of QOL-B scores, stratified by radiological severity

Mean (SD) QOL-B scores according to radiological severity (<3 lobes vs. ≥3 lobes affected / cystic bronchiectasis)			
**QOL-B scale**	**<3 lobes**	**≥ 3 lobes or cystic bronchiectasis**	***p*-Value** [1]
Respiratory Symptoms	57.8 (21.5)	55.3 (20.7)	0.058
Physical Functioning	45.2 (31.2)	39.9 (28.8)	**0.022**
Vitality	41.8 (21.2)	42.2 (21.5)	0.770
Role Functioning	61.5 (26.6)	57.3 (27.7)	**0.027**
Health Perceptions	38.1 (22.6)	35.2 (22.5)	**0.048**
Emotional Functioning	69.8 (21.8)	68.8 (21.9)	0.560
Social Functioning	61.9 (26.7)	58.7 (27.0)	0.088
Treatment Burden	52.0 (25.7)	50.9 (24.8)	0.524

[1] Differences between groups were assessed by the Mann-Whitney U test. Abbreviations: SD, standard deviation; QOL-B, Quality of Life Questionnaire-Bronchiectasis.

Supplementary Table S2.8. Discrimination of QOL-B scores, stratified by history of prior thoracic surgery

**Mean (SD) QOL-B scores for patients with prior thoracic surgery**			
**QOL-B scale**	**Yes**	**No**	***p*-Value** [1]
Respiratory Symptoms	51.3 (21.1)	56.6 (21.0)	**0.025**
Physical Functioning	37.7 (29.7)	42.2 (30.0)	0.195
Vitality	40.1 (21.1)	42.2 (21.4)	0.370
Role Functioning	53.1 (29.8)	59.4 (27.0)	0.073
Health Perceptions	33.2 (21.1)	36.6 (22.7)	0.261
Emotional Functioning	69.3 (21.8)	69.2 (21.9)	0.938
Social Functioning	51.2 (24.4)	60.6 (27.0)	**0.002**
Treatment Burden	47.3 (26.4)	51.8 (24.9)	0.196

[1] Differences between groups were assessed by the Mann-Whitney U test. Abbreviations: SD, standard deviation; QOL-B, Quality of Life Questionnaire-Bronchiectasis.
