# Supplementary material for: Psychometric Validation of the German Translation of the Quality of Life Questionnaire-Bronchiectasis (QOL-B)—Data from the German Bronchiectasis Registry PROGNOSIS
Source: J Clin Med. 2022 Jan 15;11(2):441. doi: 10.3390/jcm11020441 (PMC8781204; doi:10.3390/jcm11020441)
Supplement: Supplementary file 1 [file jcm-11-00441-s001.zip › Quellhorst_JCM_Supplementary_material_S2_05-Dec-2021.pdf]

Psychometric Validation of the German Translation of the Quality of Life Questionnaire Bronchiectasis (QOL-B) – Data from the German Bronchiectasis Registry PROGNOSIS

Laura Quellhorst, Grit Barten, Andrés de Roux, Roland Diel, Pontus Mertsch, Isabell Pink, Jessica Rademacher, Sivagurunathan Sutharsan, Tobias Welte, Annegret Zurawski, and Felix C. Ringshausen, and the PROGNOSIS study group

SUPPLEMENTARY MATERIALS 2 (S2)

Supplementary Table S2.1. Comparison of demographic and baseline characteristics between all subjects enrolled in PROGNOSIS until Mach 2018 (N=1000) and those who completed the QOL-B (N=904)

Variable		Value	Value
**N (total)**		**904**	**1000**
Age (years), mean (SD)		59.5 (15.8)	59.3 (15.8)
	<50 years, n (%)	215 (23.8)	247 (24.7)
	50-69 years, n (%)	401 (44.4)	442 (44.2)
	70-79 years, n (%)	253 (28.0)	273 (27.3)
	≥80 years, n (%)	35 (3.9)	38 (3.8)
Females, n (%)		538 (59.5)	593 (59.3)
BMI (kg/m^2), mean (SD)		24.1 (4.5)	24.1 (4.6)
	<18.5 kg/m^2	80 (8.8)	88 (8.8)
	18.5-30 kg/m^2	740 (81.9)	822 (82.2)
	>30 kg/m^2	84 (9.3)	90 (9.0)
FEV_1 %predicted, mean (SD)		69.8 (26.8)	69.4 (26.6)
	<30 %predicted, n (%)	58 (6.4)	63 (6.3)
	30-49 %predicted, n (%)	180 (19.9)	202 (20.2)

	50-79 %predicted, n (%)	322 (35.6)	360 (36.0)
	≥80 %predicted, n (%)	344 (38.1)	375 (37.5)
Etiology, n (%)			
	Idiopathic	337 (37.3)	358 (35.8)
	Postinfectious/posttuberculous	180 (19.9)	212 (21.2)
	COPD	133 (14.7)	149 (14.9)
	Asthma	99 (11.0)	111 (11.1)
	Primary Ciliary Dyskinesia / Kartagener syndrome	79 (8.7)	88 (8.8)
	Other	76 (8.4)	82 (8.2)
Radiological Severity			
	<3 lobes affected	325 (36.0)	371 (37.1)
	≥3 lobes affected	422 (46.7)	463 (46.3)
	Cystic bronchiectasis	157 (17.4)	166 (16.6)
MRC dyspnea scale, n(%)			
	1	228 (25.2)	262 (26.2)
	2	301 (33.3)	339 (33.9)
	3	222 (24.6)	236 (23.6)
	4	108 (11.9)	116 (11.6)
	5	45 (5.0)	47 (4.7)
	1-3	751 (83.1)	837 (83.7)
	4-5	153 (16.9)	163 (16.3)
Smoking, n (%)			
	Active smoker	57 (6.3)	65 (6.5)
	Former smoker	335 (37.1)	371 (37.2)
	Never smoked	512 (56.6)	560 (56.2)
Exacerbations in the past 12 months, median (IQR)		1 (0-3)	1 (0-3)
	0, n (%)	271 (30.0)	304 (30.4)
	1-2, n (%)	361 (39.9)	394 (39.4)
	≥3, n (%)	272 (30.1)	302 (30.2)
Hospitalizations in the past 12 months, median (IQR)		0 (0-1)	0 (0-1)
Prior hospital admission, n (%)		349 (38.6)	387 (38.7)
Regular pharmacological treatment of bronchiectasis		704 (77.9)	766 (76.6)
Prior thoracic surgery		79 (8.7)	90 (9.0)
Regular sputum production, n (%)		704 (77.9)	780 (78.0)
Average daily sputum volume, median (IQR)		20 (10-50)	20 (10-50)

	0 mL/day, n (%)	284 (31.4)	311 (31.1)
	1-10 mL/day, n (%)	261 (28.9)	284 (28.4)
	11-20 mL/day, n (%)	122 (13.5)	128 (12.8)
	21-50 mL/day, n (%)	151 (16.7)	180 (18.0)
	51-100 mL/day, n (%)	60 (6.6)	67 (6.7)
	>100 mL/day, n (%)	26 (2.9)	30 (3.0)
QoL-B scales, mean (SD)			
	Respiratory Symptoms (n=892)	56.2 (21.0)	
	Physical Functioning (n=889)	41.8 (29.8)	
	Vitality (n=892)	42.0 (21.4)	
	Role Functioning (n=898)	58.8 (27.4)	
	Health Perceptions (n=891)	36.3 (22.6)	
	Emotional Functioning (n=889)	69.2 (21.9)	
	Social Functioning (n=878)	59.9 (26.9)	
	Treatment Burden (n=645) [1]	51.3 (25.1)	
Microbiology		n = 680	n = 756
	Pseudomonas aeruginosa, n (%)	223 (32.8)	249 (32.9)
	Staphylococcus aureus, n (%)	112 (16.5)	124 (16.4)
	Haemophilus influenzae, n (%)	93 (13.7)	103 (13.6)
	Aspergillus fumigatus, n (%)	73 (10.7)	79 (10.4)
	Nontuberculous mycobacteria, n (%)	41 (6.0)	48 (6.3)
BSI		n = 666	n = 736
	Mild (0–4)	150 (22.5)	171 (23.2)
	Moderate (5–8)	390 (58.6)	436 (59.2)
	Severe (≥9)	126 (18.9)	129 (17.5)
Comorbidities			
	Cardiovascular	354 (39.2)	383 (38.3)
	COPD	269 (29.8)	307 (30.7)
	Asthma	269 (29.8)	293 (29.3)
	Chronic rhinosinusitis	270 (29.9)	293 (29.3)
	Gastro-esophageal reflux (self-reported)	166 (18.4)	188 (18.8)
	Nasal polyps	141 (15.6)	155 (15.5)
	Malignancy	108 (11.9)	117 (11.7)
	Osteoporosis	98 (10.8)	104 (10.4)
	Depression	86 (9.5)	99 (9.9)

	Diabetes	79 (8.7)	88 (8.8)
	Renal insufficiency	71 (7.9)	73 (7.3)
	Anxiety disorder	34 (3.8)	39 (3.9)
	Liver cirrhosis	9 (1.0)	11 (1.1)

[1] Patients not receiving bronchiectasis treatment were instructed to skip the Treatment Burden scale. Abbreviations: BMI, body mass index; BSI, Bronchiectasis Severity Index; COPD, chronic obstructive pulmonary disease; FEV_1, forced expiratory volume in 1 second; IQR, interquartile range; MRC, Medical Research Council; QOL-B, QOL-B, Quality of Life Questionnaire-Bronchiectasis; PCD, primary ciliary dyskinesia; SD, standard deviation.

Supplementary Table S2.2. Discrimination of QOL-B scores, stratified by MRC dyspnea scale

**Mean (SD) QOL-B scores at baseline according to MRC dyspnea scale**						
**QOL-B scale**	**1**	**2**	**3**	**4**	**5**	***p*-Value** [1]
Respiratory Symptoms	69.5 (19.0)	56.9 (17.7)	49.6 (18.4)	42.1 (17.9)	42.4 (21.2)	<0.001
Physical Functioning	67.4 (27.4)	44.5 (23.5)	27.8 (18.9)	15.9 (18.5)	12.3 (21.8)	<0.001
Vitality	51.5 (21.3)	43.1 (20.7)	38.9 (18.9)	31.0 (18.5)	29.0 (22.4)	<0.001
Role Functioning	73.9 (23.3)	64.1 (23.4)	52.9 (22.2)	32.9 (21.7)	25.5 (21.4)	<0.001
Health Perceptions	51.0 (23.9)	37.9 (19.9)	29.0 (18.0)	22.7 (16.4)	19.0 (16.4)	<0.001
Emotional Functioning	74.9 (20.7)	71.8 (20.7)	66.1 (20.5)	59.3 (20.8)	53.3 (26.7)	<0.001
Social Functioning	66.3 (26.8)	62.1 (22.8)	54.4 (27.4)	48.9 (26.3)	47.8 (26.3)	<0.001
Treatment Burden	66.3 (22.7)	54.8 (22.9)	44.6 (23.0)	36.3 (23.1)	32.9 (21.8)	<0.001

[1] Differences between groups were assessed by the Kruskal-Wallis test. Abbreviations: MRC, Medical Research Council; QOL-B, Quality of Life Questionnaire-Bronchiectasis; SD, standard deviation.

Supplementary Table S2.3. Discrimination of QOL-B scores, stratified by BSI categories (n = 666)

**Mean (SD) QOL-B scores at baseline according to BSI category**				
**QOL-B scale**	**Mild (0–4)**	**Moderate (5–8)**	**Severe (≥9)**	***p*-Value** [1]
Respiratory Symptoms	61.8 (18.7)	52.3 (21.5)	51.4 (18.0)	<0.001
Physical Functioning	56.9 (27.6)	35.6 (28.7)	32.5 (26.0)	<0.001
Vitality	46.2 (20.9)	40.0 (21.9)	38.9 (19.5)	0.002
Role Functioning	72.5 (21.4)	53.7 (27.3)	46.5 (26.1)	<0.001
Health Perceptions	43.2 (22.4)	31.8 (21.3)	29.9 (19.4)	<0.001
Emotional Functioning	72.8 (20.2)	66.6 (22.8)	69.7 (20.4)	0.025
Social Functioning	61.8 (25.1)	57.0 (27.8)	53.5 (26.3)	0.033
Treatment Burden	55.1 (23.6)	46.9 (24.6)	45.1 (22.1)	0.006

[1] Differences between groups were assessed by the Kruskal-Wallis test. Abbreviations: BSI, Bronchiectasis Severity index; QOL-B, Quality of Life Questionnaire-Bronchiectasis; SD, standard deviation.

Supplementary Table S2.4. Discrimination of QOL-B scores, stratified by $ppFEV_1$ (categorized)

Mean (SD) QOL-B scores at baseline according to $ppFEV_1$					
**QOL-B scale**	**≥80**	**50-79**	**30-49**	**<30**	***p*-Value** [1]
Respiratory Symptoms	63 (18.8)	54.0 (20.2)	50.2 (19.1)	42.6 (18.6)	<0.001
Physical Functioning	55.5 (28.7)	40.4 (26.1)	25.3 (22.3)	15.2 (16.4)	<0.001
Vitality	45.2 (21.5)	41.5 (21.3)	41.5 (19.9)	31.9 (19.5)	<0.001
Role Functioning	66.3 (24.1)	59.5 (24.0)	51.1 (27.7)	33.4 (27.0)	<0.001
Health Perceptions	42.2 (23.6)	36.2 (20.7)	30.3 (20.4)	25.4 (19.2)	<0.001
Emotional Functioning	71.1 (21.3)	70.3 (20.4)	67.4 (22.3)	58.5 (23.2)	0.003
Social Functioning	60.5 (25.7)	57.4 (27.4)	60.1 (25.8)	51.7 (25)	0.087
Treatment Burden	59.2 (24.3)	49.9 (24.8)	46.6 (23.5)	38.8 (23.9)	<0.001

[1] Differences between groups were assessed by the Kruskal-Wallis test. Abbreviations: $ppFEV_1$, forced expiratory volume in one second (% predicted); QOL-B, Quality of Life Questionnaire-Bronchiectasis; SD, standard deviation.

Supplementary Table S2.5. Discrimination of QOL-B scores, stratified by average daily sputum volume (categorized)

Mean (SD) QOL-B scores at baseline according to average daily sputum volume (mL/day)							
**QOL-B scale**	**0**	**1-10**	**11-20**	**21-50**	**51-100**	**>100**	***p*-Value** [1]
Respiratory Symptoms	61.9 (22.6)	57.0 (19.4)	52.1 (17.8)	50.8 (18.1)	43.1 (17.4)	39.8 (14.7)	<0.001
Physical Functioning	44.5 (31.4)	42.0 (28.4)	37.8 (26.3)	37.7 (28.4)	30.7 (24.4)	27.0 (23.5)	0.001
Vitality	44.0 (22.1)	43.4 (21.0)	39.5 (19.7)	41.4 (20.2)	34.0 (20.1)	38.2 (23.7)	0.059
Role Functioning	62.3 (27.8)	57.4 (28.0)	55.8 (21.9)	57.3 (26.8)	44.2 (27.0)	52.1 (20.5)	<0.001
Health Perceptions	41.4 (23.4)	36.8 (22.9)	32.5 (19.2)	33.5 (20.8)	24.1 (19.0)	23.4 (15.0)	<0.001
Emotional Functioning	70.5 (22.3)	69.3 (20.9)	67.3 (20.0)	68.8 (21.9)	62.1 (21.3)	62.0 (21.9)	0.056
Social Functioning	65.1 (26.5)	60.1 (25.2)	55.5 (25.0)	55.1 (26.3)	43.3 (27.6)	46.0 (23.4)	<0.001
Treatment Burden	58.8 (24.4)	52.1 (24.9)	49.4 (23.7)	46.8 (24.0)	39.0 (24.3)	35.4 (22.7)	<0.001

[1] Differences between groups were assessed by the Kruskal-Wallis test. Abbreviation: QOL-B, Quality of Life Questionnaire-Bronchiectasis; SD, standard deviation.

Supplementary Table S2.6. Discrimination of QOL-B scores, stratified by regular pharmacological treatment of bronchiectasis

Mean (SD) QOL-B scores for patients with regular pharmacological treatment			
**QOL-B scale**	**Yes**	**No**	***p*-Value** [1]
Respiratory Symptoms	54.3 (20.6)	62.7 (21.4)	<0.001
Physical Functioning	40.1 (29.0)	48.1 (31.9)	0.003
Vitality	41.7 (21.4)	43.2 (21.4)	0.422
Role Functioning	57.2 (27.4)	64.7 (26.6)	0.001
Health Perceptions	35.2 (22.3)	40.2 (23.2)	0.008
Emotional Functioning	69.1 (21.9)	69.5 (21.7)	0.906
Social Functioning	57.8 (27.1)	67.0 (24.9)	<0.001
Treatment Burden	49.5 (25.0)	61.2 (23.4)	<0.001

[1] Differences between groups were assessed by the Mann-Whitney U test. Abbreviations: QOL-B, Quality of Life Questionnaire-Bronchiectasis; SD, standard deviation.

Supplementary Table S2.7. Discrimination of QOL-B scores, stratified by radiological severity

Mean (SD) QOL-B scores according to radiological severity (<3 lobes vs. ≥3 lobes affected / cystic bronchiectasis)			
**QOL-B scale**	**<3 lobes**	**≥ 3 lobes or cystic bronchiectasis**	***p*-Value** [1]
Respiratory Symptoms	57.8 (21.5)	55.3 (20.7)	0.058
Physical Functioning	45.2 (31.2)	39.9 (28.8)	**0.022**
Vitality	41.8 (21.2)	42.2 (21.5)	0.770
Role Functioning	61.5 (26.6)	57.3 (27.7)	**0.027**
Health Perceptions	38.1 (22.6)	35.2 (22.5)	**0.048**
Emotional Functioning	69.8 (21.8)	68.8 (21.9)	0.560
Social Functioning	61.9 (26.7)	58.7 (27.0)	0.088
Treatment Burden	52.0 (25.7)	50.9 (24.8)	0.524

[1] Differences between groups were assessed by the Mann-Whitney U test. Abbreviations: SD, standard deviation; QOL-B, Quality of Life Questionnaire-Bronchiectasis.

Supplementary Table S2.8. Discrimination of QOL-B scores, stratified by history of prior thoracic surgery

**Mean (SD) QOL-B scores for patients with prior thoracic surgery**			
**QOL-B scale**	**Yes**	**No**	***p*-Value** [1]
Respiratory Symptoms	51.3 (21.1)	56.6 (21.0)	**0.025**
Physical Functioning	37.7 (29.7)	42.2 (30.0)	0.195
Vitality	40.1 (21.1)	42.2 (21.4)	0.370
Role Functioning	53.1 (29.8)	59.4 (27.0)	0.073
Health Perceptions	33.2 (21.1)	36.6 (22.7)	0.261
Emotional Functioning	69.3 (21.8)	69.2 (21.9)	0.938
Social Functioning	51.2 (24.4)	60.6 (27.0)	**0.002**
Treatment Burden	47.3 (26.4)	51.8 (24.9)	0.196

[1] Differences between groups were assessed by the Mann-Whitney U test. Abbreviations: SD, standard deviation; QOL-B, Quality of Life Questionnaire-Bronchiectasis.
